# Silicene, germanene and other group IV 2D materials

**DOI:** 10.3762/bjnano.9.248

**Published:** 2018-10-10

**Authors:** Patrick Vogt

**Affiliations:** 1 Institut für Physik, Technische Universität Chemnitz, Reichenhainer Str. 70, Chemnitz, Germany

**Keywords:** 2D materials, germanene, silicene

The discovery of graphene and its tremendous impact on scientific research has initiated the search for other elemental two-dimensional (2D) honeycomb materials with potentially similar exotic properties, as predicted by theoretical investigations. These properties may allow the application of these layered structures in novel electronic devices, including ultrafast electronics, spintronics, sensors, and novel device concepts exploiting their topological properties. In recent years this search has lead to the discovery of other members of this family of 2D materials based on other group IV elements.

In 2012 silicene was first synthesized under ultrahigh vacuum conditions on a silver(111) single crystal by Si molecular beam epitaxy (MBE) [1–2] and at around the same time on zirconium diboride thin films grown on Si(111) substrates by Si segregation through the film [3]. The synthesis of silicene further launched an intensive search for other 2D elemental materials synthesized under ultrahigh vacuum by MBE-like methods. The synthesis of germanene (2D germanium) was reported in 2014 [4] and the synthesis of stanene (2D tin) in 2016 [5].

Except for their 2D character, these materials are substantially different from prototypical graphene. First of all, these materials do not exist in nature, nor do their 3D layered parent crystals from which single layers can be exfoliated. From this it follows that these materials have to be synthesized either chemically or by epitaxial growth on a supporting substrate. Thus, it should be considered that the substrate could influence the structural and electronic properties of the 2D layer.

**Figure 1:**
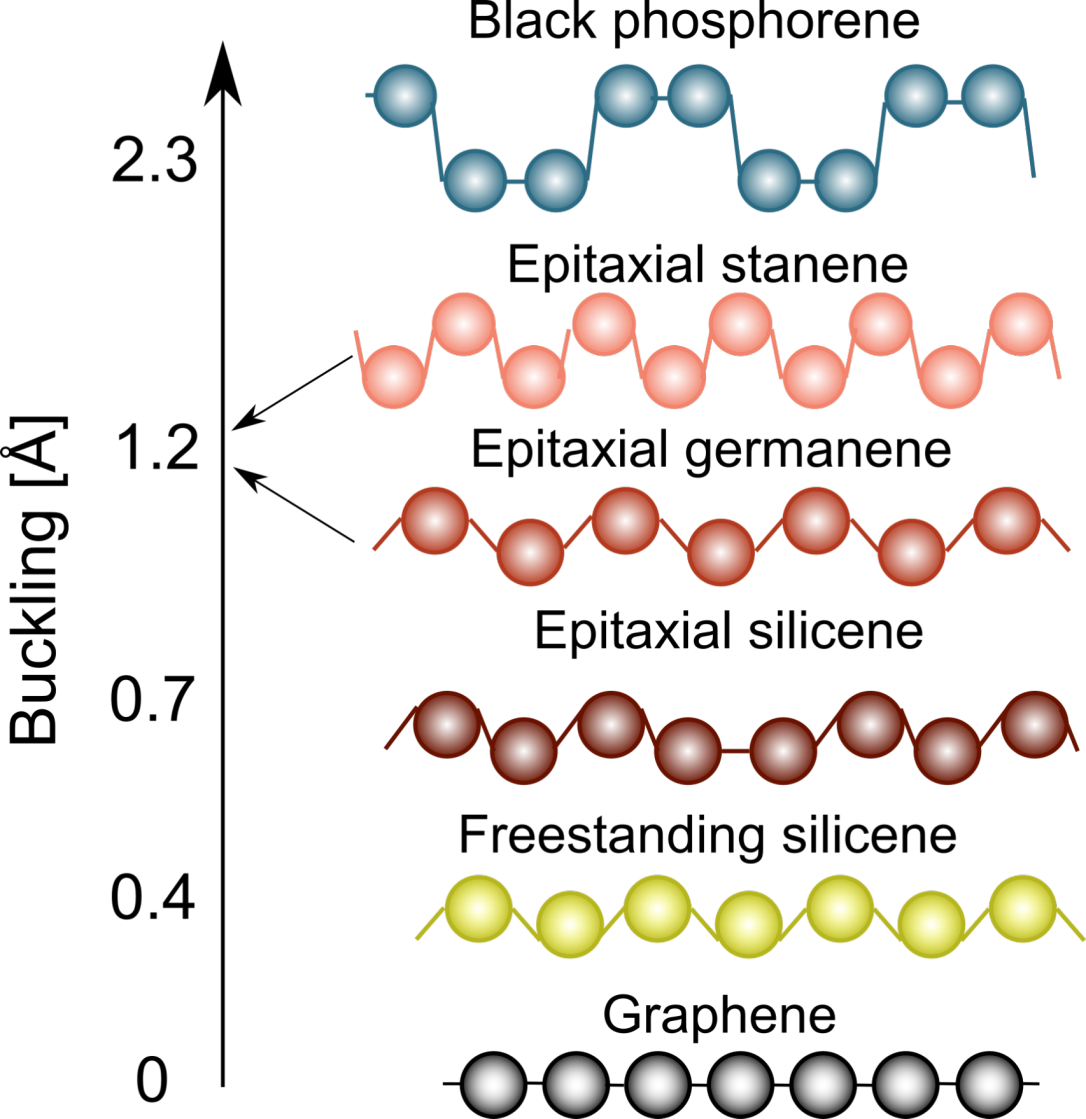
Illustration of the increasing buckling of different elemental 2D materials.

Secondly, of these materials, only graphene is fully flat, which results from the pure sp^2^ hybridization of its constituting carbon atoms. On the contrary, other group IV elements energetically prefer hybridization with an additional sp^3^ character when forming a 2D honeycomb layer, which increases with increasing atomic size. As a consequence of this mixed hybridization, the bonds between adjacent atoms of the lattice are buckled, resulting in a layer that is not completely flat. Such buckling within the layer is illustrated in Figure 1 for different elemental 2D materials. For the case of silicene, it can also be seen here that the substrate material further influences the buckling within the 2D layer, resulting from the interaction between the two systems. Free-standing silicene has a buckling around 0.44 Å [6], while epitaxial silicene on Ag(111) has a value of 0.75 Å [1].

The buckling and the significant influence of the substrate have been considered as disadvantages since they influence the properties of the 2D layer. On the other hand, both characteristics also offer possibilities for tuning the properties of the 2D layer. External stimuli or the choice of the substrate can effect the buckling, which in turn, alters the properties of the 2D layer. The related modification might include, for example, the tunability of the electronic band gap, modification of the electronic band structure, or tuning the 2D topological properties. Some of these external influences might include:

- the substrate material
- external electric or magnetic fields
- tensile or compressive strain
- functionalization by atomic or molecular species

This means that the apparent disadvantage of these materials to develop a (low) buckling is in fact an advantage since it facilitates control of the 2D layer properties, for example, via chemical functionalization or external fields. This could be efficiently utilized in a transistor, where the electronic band gap can then be tuned by the electric field applied perpendicular to the lattice plane. As an example, ab initio calculations have shown that the two sub-lattices in silicene, resulting from the buckling, are moved further apart by an orthogonal electric field, which leads to a band gap opening [7–8].

Another important advantage of these new materials is the significant spin–orbit interaction, which also increases with increasing atomic size of the involved elements. This opens the way to observe a quantum spin Hall effect, for example, in germanene or stanene in an accessible temperature range, possibly even at room temperature. The occurrence of topologically nontrivial properties will be more robust for the heavier constituting elements because of the related stronger spin–orbit interaction. Topological properties are expected to enable entirely new concepts in electronic devices.

These characteristics make the young class of buckled 2D elemental materials a new progressing research field with anticipated outstanding properties of their members or as a result of their modification. In all these cases, the buckled atomic structure and the significant spin–orbit interaction may play a key role in the development of these properties.

However, before such applications are realized and final products find their way to the market, numerous technical and fundamental issues must first be solved. This concerns the synthesis of these materials, a deeper understanding of their physical properties, as well as the modification of these materials according to the factors mentioned above. Such knowledge will also help to understand the properties of 2D layers composed of elements that do not belong to group IV of the periodic table, for example, borophene [9], blue phosphorous (phosphorene) [10], or antimonene [11], most of which also show buckling.

The aim of this Thematic Series was to address the theoretical background, the growth and synthesis, the properties and application and the related difficulties still to overcome related to this new class of elemental group IV 2D materials. Of course, the included contributions can only present a snapshot of all the activities in this growing research field. I hope that the choice of articles is inspiring to the readers and reflects the important aspects of this research. I thank all authors and colleagues that have contributed their intriguing results to this Thematic Series.

Patrick Vogt

Chemnitz, September 2018
